# MicroRNA-Induced Regulation of the IGF-1 System in Various Types of Cancer

**DOI:** 10.3390/genes16101135

**Published:** 2025-09-25

**Authors:** George Triantafyllou, Mary Kategianni, Maria Maridaki, Michael Koutsilieris, Anastassios Philippou

**Affiliations:** 1 Department of Anatomy, Medical School, National and Kapodistrian University of Athens, 11527 Athens, Greece; georgerose406@gmail.com; 2 Department of Physiology, Medical School, National and Kapodistrian University of Athens, 11527 Athens, Greece; mkategianni@gmail.com (M.K.); mkoutsil@med.uoa.gr (M.K.); 3 School of Physical Education and Sport Science, National and Kapodistrian University of Athens, 17237 Athens, Greece; mmarida@phed.uoa.gr

**Keywords:** microRNAs, IGF-1, cancer biology, biomarker, molecular pathways

## Abstract

Insulin-like growth factor 1 (IGF-1) is an important endocrine and autocrine/paracrine factor that regulates various cellular responses in multiple biological systems. Its actions are mediated mainly via its binding to the type 1 IGF receptor (IGF-1R), while its bioactivity is also modulated by the IGF-binding proteins (IGFBPs). The IGF-1 system regulates cell growth, differentiation and energy metabolism and thus plays a crucial role in the modulation of key aspects of cancer biology, such as cancer cell growth, survival, transformation and invasion. The synthesis of IGF-1 is regulated, among other factors, by microRNAs (miRNAs), and it has been shown that the miRNA-induced regulation of IGF-1 is implicated in various stages of tumor development and/or progression in different types of cancer. The aim of this review was to identify and characterize the miRNA-induced regulation of the IGF-1 system in various types of cancer. It was revealed that many miRNAs can be used as potential biomarkers, while others may contribute to metastasis regulation, targeting components of the IGF-1 bioregulation system and being implicated in cancer staging and/or progression. Additional miRNAs and their role in IGF-1’s effects on other types of cancer have also been identified. Nevertheless, future studies are needed to expand the current knowledge on the role of miRNAs in the regulation of other components of the IGF-1 bioregulation system and in various types of cancer, contributing further to the characterization of the role of miRNAs and their target genes as pathogenic, therapeutic and diagnostic molecules for cancer in clinical practice.

## 1. Introduction

Insulin-like growth factor 1 (IGF-1) is an endocrine and autocrine/paracrine peptide protein similar in function and structure to proinsulin [1,2], playing a significant role in multiple physiological processes [3,4] and predominant in embryonic development and postnatal growth [5]. The liver is the main source of IGF-1, while other organs including the brain, kidneys and muscles secrete IGF-1 in lower levels [6]. IGF-1 acts mainly via type 1 receptor (IGF-1R), a transmembrane tyrosine kinase receptor, which mediates IGF-1 signaling [7].

IGF-1’s actions are implicated in many physiological processes. It is a major factor in muscle cell growth, differentiation and regeneration [5] and in bone formation and metabolism, having anabolic action on osteoblasts [8,9]. In addition, its role is expanded in other tissues such as the mammary tissue, in the central nervous system (CNS) and the immune system, and in processes including development, ageing and cancer [10,11,12,13,14,15,16].

The *IGF1* gene is located in the long arm of chromosome 12 [17], and it is highly conserved [18]. Multiple mRNAs are produced from the *IGF1* gene as a result of its various transcription initiation sites, alternative splicing and different polyadenylation sites [18,19,20]. Moreover, post-transcriptional regulation processes, which interfere with mRNA spicing, maturation, transport, turnover and translation, are regulated, among other factors, by microRNAs and RNA-binding proteins (RBPs), while many of them have been shown to regulate the expression of IGF-1 and IGF-1R [21]. Interestingly, the 3′-untranslated region (3′-UTR) of IGF-I contains 11 of the top 50 conserved miRNA sites identified [22].

## 2. MicroRNA-Induced Regulation of IGF-1

**Figure 1:**
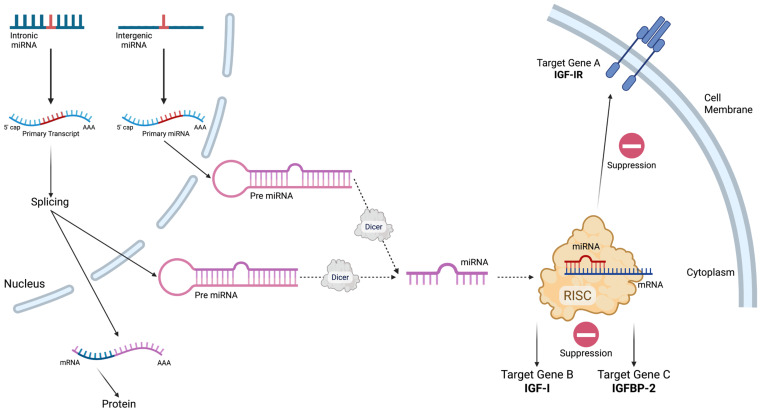
A schematic representation of the biogenesis and function of the microRNAs (miRNAs). MiRNAs can be derived from intronic regions of protein-coding genes (intronic miRNA) or intergenic regions (intergenic miRNA). After transcription, the primary miRNA (Pre miRNA) is processed into primary miRNA in the nucleus. Pre miRNAs are exported to the cytoplasm, where the enzyme Dicer further processes them into mature miRNA. The miRNA is incorporated into the RNA-induced silencing complex (RISC). Moreover, the RISC–miRNA complex binds to target messenger RNAs (mRNAs) to suppress target gene expression. The figure depicts examples of target genes suppressed by miRNAs, including IGF-IR (Target Gene A), IGF-I (Target Gene B) and IGFBP-2 (Target Gene C).

MicroRNAs (miRNAs, miRs) are a class of endogenous, small, non-coding RNAs which interfere with the translation of complementary mRNAs in a sequence-specific mechanism (Figure 1).

They are approximately 22 nucleotides long and consist of some of the most abundant molecules regulating gene expression [23]. Originally identified in *C. elegans* [24], they appear to be active in all eukaryotes, and their action affects gene expression, varying considerably—from cell death to cell differentiation—in different organisms [23]. A variety of miRNAs that target the IGF-1 transcription products have already been identified, some of which are tissue- or age-specific [25].

A particular group of miRNAs that show great specificity in muscle tissue are called myomiRs. The members of this group are miR-1, miR-133, miR-206, miR-208b, miR-499 and miR-486 [26]. They are preferentially expressed in cardiac and skeletal muscle tissue and are considered to significantly affect muscle phenotype and physiology [27]. Furthermore, IGF-1 has a substantial role in bone formation and remodeling, with miRNAs contributing to this pathway. For example, miR-29 is a molecule that contributes to osteoblastogenesis and osteoblast differentiation by targeting several molecules, while its expression has been linked with differentiation, reduced cell proliferation and senescence in many cell types [28]. It is well established that the loss of function of the IGF-1 bioregulation system is of great significance in ageing and the regulation of life span in several organisms, and many signaling proteins have been associated with the IGF-1 pathway(s) [29,30]. A study on long-lived mutant mice showed that the profile of miRNA expression changes during ageing. Specifically, three miRNAs were identified to be upregulated, miR-470, miR-669b and miR-681, while they were mainly localized at the hippocampus. Expression of miRNAs is important in pathological conditions, as their expression appears to change in tumors, suggesting their contribution to tumorigenesis [31,32]. Interestingly, early studies have shown that microRNAs can be transferred between different cell types through gap junctions or intracellular contact, which gives a potential new approach for considering microRNAs as therapeutic targets [33].

Moreover, the oncogenic potential of IGF-1 regulation is mediated via physiologic signaling pathways, mainly PI3K/AKT/mTOR and RAS/RAF/MEK/ERK [34,35]. Multiple miRNAs discussed in the present review can modulate the expression of genes (signaling factors) in the IG-1 downstream pathways and exhibit tumor-suppressive functions [34,35].

## 3. MicroRNA-Induced Regulation of IGF-1: Breast Cancer

The IGF-1 bioregulation system is essential for the normal development and function of the mammary gland [36], and it is also involved, along with the estrogens, in breast development [37], as well as in breast cancer [38]. It is important to mention that metastatic cancer cells secrete insulin-like growth factor binding protein-2 (IGFBP-2), a biomarker of human metastasis, which makes IGF-1 available in endothelial cells and activates the IGF-1 pathway. Through this activation, the recruitment, metastasis and angiogenesis of endothelial cells are affected. miR-126, a microRNA that suppresses metastatic endothelial recruitment, targets, among others, IGFBP-2, and it was found to be downregulated in several cancer types, thus promoting the metastatic phenotype. Moreover, when breast cancer cells were co-injected with endothelial cells, the decrease in miR-126 levels was reversed, possibly due to cellular interactions. This finding not only indicates that the IGF-1 signaling pathway plays an important role in the initiation of metastasis but also emphasizes that the microenvironment and cellular communications are of paramount importance [39,40].

Specifically, it was revealed that miR-122 directly targets the 3′-UTR of IGF-1R in breast cancer cells and limits cell proliferation as well as tumor formation and progression. Four cancer cell lines were studied, all of which presented decreased expression of miR-122 when compared to breast epithelial cells [38]. Furthermore, MCF-7 breast cancer cells were infected with miR-122 to over-express miR-122. Cell proliferation, colony formation, tumor volume, size and weight presented significantly lower values in the cells over-expressing miR-122 compared with the cells infected with an empty virus vector, which indicated that this microRNA has a key role in tumorigenesis and tumor progression [38]. Further studies confirmed that miR-122 targets the 3′-UTR of IGF-1R after examining IGF-1R expression in cells with a mutation in the 3′-UTR of IGF-1R compared to normal cells, in the presence and absence of miR-122. Supporting those findings, IGF-1R knockdown cells showed a similar proliferation rate when compared to cells over-expressing miR-122. Also, human samples from breast cancer tissue exhibited reduced miR-122 and increased IGF-1R expression when compared to samples from normal tissue, suggesting that miR-122 acts directly on IGF-1R [38]. Moreover, three more miRNAs (miR-215, miR-200 and miR-141) were identified as being downregulated in breast cancer [39], and it was proposed that these miRNAs could potentially act for an anti-neoplastic transformation of tumor cells [39].

Some breast cancer cells over-express the *ERBB2* gene, which encodes the HER2 protein (HER2-positive breast cancer cells). The use of antibodies is a widespread strategy in the treatment of several cancers including breast cancer. Although the humanized monoclonal anti-HER2 antibody Herceptin has been effectively used in cancer therapy, most of the patients that respond to therapy develop resistance to Herceptin after a year of treatment. Moreover, an upregulation of IGF-1R has been observed in those resistant cells, and there is speculation that the IGF-1 bioregulation system might be involved in the limited response to Herceptin. In this context, a microRNA predicted to target IGF-1R is miR-375, and studies have revealed that it targets the 3′-UTR of the IGF-1R mRNA [41]. Herceptin-resistant cells were co-transfected with constructs containing the wild type or mutant 3′-UTR of IGF-1R and either a vector expressing miR-375 mimics or a control vector. It was shown that IGF-1R expression was lower in the cells containing the wild-type 3′-UTR in the presence of miR-375, while the mutant cells’ expression was not affected, indicating that miR-375 targets the 3′-UTR of IGF-1R. Moreover, over-expression of miR-375 decreased the IGF-1R levels compared to those in control cells, while cells treated with miR-375 inhibitor expressed higher levels of IGF-1R. Additionally, IGF-1R expression was found to decrease as an effect of the increased expression of miR-375 [40], while miR-375 was found to be downregulated in Herceptin-resistant cells. Moreover, in these studies, SKBr-3 breast cancer cells were cultured in the presence of Herceptin for six months to become Herceptin-resistant. When miR-375 was over-expressed and, therefore, IGF-1R levels decreased, the sensitivity of the cells to therapy increased [40]. Contrariwise, when miR-375 was inhibited, the cells became less responsive. Overall, these findings indicate that there is an underlying mechanism that connects higher IGF-1R expression levels with cell resistance to Herceptin. Animal studies led to similar results: Over-expression of miR-375 in mice injected with breast cancer cells to develop tumors had a positive effect on therapy response. The tumor weight and volume were lower, and mouse survival was increased [40].

Lastly, another microRNA studied in breast cancer is miR-145. This microRNA also targets IGF-1R, among other molecules. Transfection of breast cancer cells with miR-145 mimics resulted in reduced cell growth and mobility, while studies in patients’ tissues also led to similar results [42].

## 4. MicroRNA-Induced Regulation of IGF-1: Gastric and Colorectal Cancer

In a study using gastric cancer cells and tissues, miR-7 was found to be downregulated specifically in metastatic cells and tissues, while IGF-1R was recognized as a direct target of miR-7 [43]. In further experiments, it was shown that upregulation of miR-7 led to a less metastatic phenotype with lower invasion and migration levels observed both in vitro and in vivo. On the other hand, miR-7 downregulation increased the rate of these processes. As far as the effect on IGF-1R is concerned, small interfering RNA (siRNA) was used to silence IGF-1R and, as expected, metastasis decreased in accordance with the previous findings, indicating that miR-7 can inhibit metastasis [43].

Colorectal cancer (CRC) is one of the most common types of cancer worldwide and is characterized by poor prognosis and treatment. Many studies have been conducted aiming to elucidate the underlying pathophysiological mechanisms and to identify possible biomarkers or treatment targets. Towards this end, many microRNAs have been studied, and it has been shown that some of them target effectors of the IGF-1 pathway(s) and mainly IGF-1R. Using microarrays, a comparison of several microRNAs’ expression levels in colorectal cancer cells and normal tissue revealed that miR-195 and miR-497 are more than two-fold downregulated in cancerous cells [41]. Similar results were obtained when the expression levels of these two microRNAs were measured using q-PCR in six different colon cancer cell lines in parallel with a normal colon epithelial cell line [41]. Computer analysis revealed that one of the putative targets of miR-195 and miR-497 is the 3′-UTR of IGF-1R [41]. To confirm this finding, cells expressing those microRNAs were transfected with a normal or mutated fragment of the 3′-UTR of the *IGF-1R* gene, which was inserted in a vector followed by a firefly luciferase reporter gene. The IGF-1R expression was lower only in the cells with the normal IGF-1R sequence [43]. Moreover, after the addition of anti-miRs (anti-microRNA for each microRNA), only anti-miR-497 significantly increased the luciferase activity, showing that this microRNA targets the 3′-UTR directly. In the same direction, miR-497 mimics decreased the luciferase activity. Western blotting analysis for the detection of IGF-1R protein levels in colon cancer tissue also led to congruent results. The samples with high miR-497 expression had lower IGF-1R protein levels, and the colorectal cancer samples expressed higher levels of IGF-1R compared to normal tissue. This could be explained by the fact that miR-497 is downregulated in colon cancer, as already mentioned [41]. Other studies showed that miR-497 over-expression makes colon cancer cells more sensitive to apoptosis, partly due to IGF-1R downregulation [41]. These findings could be used in improving colon cancer treatment, especially since IGF-1R is a molecule already recognized to play an important role in several types of cancer.

MiR-143 is another microRNA studied in colon cancer patients, and it was found to be downregulated in blood and tumor tissue samples from colon cancer patients compared to those from healthy individuals. Interestingly, miR-143 levels were found to be lower in patients with advanced cancer compared to those at early stages, as well as in patients with lymph node metastasis compared to those without metastasis. These findings indicate not only that miR-143 might be a possible biomarker for colon cancer but also that it may provide information about cancer staging [44]. In in silico studies, IGF-1R was found to be a putative target of miR-143. Using a vector with luciferase as a reporter gene, normal or mutant 3′-UTR IGF-1R was cloned in cell cultures. After forced expression of either a control or miR-143, it was shown that miR-143 targets the 3′-UTR of IGF-1R and reduces IGF-1R protein levels. In patients’ tissues, IGF-1R levels were measured in parallel with miR-143 expression levels. It was observed that as the levels of miR-143 lowered, the levels of IGF-1R were raised, indicating that IGF-1R regulation from miR-143 is maintained in patients’ tissue. Moreover, significant differences were detected when tumors from mice over-expressing miR-143 were compared to tumors from mice with a normal microRNA expression profile. The angiogenesis, weight and volume of the mice over-expressing miR-143 were significantly lower, as were the IGF-R protein levels [44]. Furthermore, it was speculated that IGF-1R plays a role in the resistance of colon cancer cells to drugs and especially to oxaliplatin. Using a human CRC cell line over-expressing miR-143, cells were treated with different concentrations of oxaliplatin, and it was shown that cell sensitivity to chemotherapy increased when miR-143 was over-expressed. This effect was reversed when IGF-1R was also over-expressed, thus compensating for the regulatory effect of miR-143, and cell viability was equal to that of the control cells [44].

Other microRNAs studied in colorectal cancer are miR-139 and miR-145. The latter targets IGF-1R [45,46], while miR-139 was found to be downregulated in samples of CRC tissues compared to healthy tissues. In addition, it was revealed that miR-139 also targets IGF-1R, and low levels of miR-139 are associated with cancer progression and metastasis. Moreover, upregulation of miR-139 in colon cancer cells suppresses metastasis and invasion, indicating that this microRNA could act as a regulator and potential suppressor of cancer metastasis [46].

It is worth mentioning that extended inflammation may progress to tumorigenesis. Patients with inflammatory bowel disease or ulcerative colitis have a greater risk of colon cancer manifestation because their cells are in an inflammatory environment and undergo repeated cycles of cell injury and repair. In such mouse models, miR-223 was found to target IGF-1R, and over-expression of this microRNA reduced IGF-1R levels. Based on the various microRNAs studied, it was concluded that microRNA and, consequently, IGF-1 pathway deregulation might be important for the cell transition from chronic inflammation to cancer [35,47].

## 5. MicroRNA-Induced Regulation of IGF-1: Lung Cancer

MiR-486 has been related to lung cancer and been proposed as a potential biomarker for the detection of lung cancer. Studies conducted in non-small cell lung cancers (NSCLCs) showed that miR-486 was greatly downregulated as compared to normal tissues [48].

To further investigate the role of miR-486 in lung cancer cells, it was over-expressed in two different lung cancer cell lines, H460 and A549. It was found that miR-486 upregulation resulted in reduced cell proliferation and colony formation, indicating that miR-486 decreased cell growth in both cell lines [48]. Another interesting finding was that, when miR-486 was over-expressed in these two cell lines, the IGF-1R protein levels were significantly reduced, while the mRNA levels did not present a corresponding reduction. As far as the target of miR-486 is concerned, in silico analyses revealed IGF-1R as a putative target, among others. To verify those findings, normal and mutant 3′-UTRs of IGF-1R were inserted into a vector with a luciferase reporter gene and then cloned to A549 cancer cells. As expected, the cells with the normal 3′-UTR of IGF-1R presented lower luciferase activity, suggesting that miR-486 is complementary to a sequence in the 3′-UTR of IGF-1R. In vivo studies also showed similar results; H460 and A549, either over-expressing miR-486 or not, were injected into mice, and the tumor volume and IGF-1R expression levels were significantly lower in mice over-expressing miR-486 [48]. Interestingly, A549 cells over-expressing miR-486 did not form any tumors, indicating that this microRNA contributes to tumor suppression.

Another microRNA studied in lung cancer is miR-335, especially in small cell lung cancer, which is a rapidly progressing type of cancer. Specifically, in SBC-5 cells, miR-335 was found to be downregulated, exhibiting a phenotype of extended bone damage after metastasis. When miR-335 was over-expressed, it prevented extended cell proliferation and migration and resulted in reduced colony formation and fewer osteolytic lesions. Moreover, miR-335 was found to target IGF-1R and reduce its translation. Thus, it was suggested that the downregulation of miR-335 promotes a metastatic phenotype with bone invasion through the deregulation of IGF-1R [49].

## 6. MicroRNA-Induced Regulation of IGF-1: Hepatic and Pancreatic Cancer

Apart from its role in muscle, breast and colorectal cancer, miR-145 has also been studied in hepatocellular carcinoma. Its levels were found to be significantly reduced in cancer cells, and in silico analysis revealed that the 3′-UTR of IGF-1R was among the predicted putative targets of the IGF-1 pathway. Luciferase reporter assay verified that the 3′-UTR is a direct target of miR-145 in hepatocellular carcinoma cells [50]. This indicated that miR-145 may play a universal role in many different cancer cells and, thus, it could be widely used in cancer treatment.

Patients with hepatocellular carcinoma often develop insulin resistance, which deteriorates the prognosis of the disease and survival. It has been speculated that IGF-1 acts positively on patients with insulin resistance, as it exerts insulin-like activity in regulating glucose uptake by tissues, and it has been shown that treatment with recombinant IGF-1 can increase sensitivity to glucose [51]. Decreased levels of IGF-1 have been detected in hepatocellular carcinoma tissues. In addition, high levels of miR-190 have been detected in cancer tissues, and as miR-190b expression increases, IGF-1 expression decreases. In addition, in silico analysis pointed out IGF-1 as a possible target of miR-190b. Sequential experiments including luciferase reporter assay, quantitative PCR, Western blotting analysis and immunofluorescence revealed the 3′-UTR of IGF-1 as a direct target of miR-190b [52]. Moreover, a functional relationship was observed, as miR-190b over-expression lowered IGF-1 levels, and downregulation of miR-190b led to higher IGF-1 expression. Also, low IGF-1 levels were associated with insulin resistance, and restoration of IGF-1 levels had a positive effect on IGF-1 signaling [52]. Moreover, miR-28-5p was identified as a potential biomarker of hepatocellular carcinoma, while IGF-1 and miR-28-5p had an inverse relation in cancer cells [53].

IGF-1R has also been implicated in pancreatic cancer, and it has been speculated that it is regulated by several microRNAs including miR-7, miR-139, miR-145 and miR-1 [54]. Also, IGF-1R has been recognized as a direct target of miR-497, which is detected in low levels in pancreatic cancer cells, while miR-497 inhibits cell proliferation and downregulates IGF-1R by targeting the 3′-UTR [55]. MiR-100 was more thoroughly studied in five pancreatic cell lines, two metastatic (S2VP10 and S2CP9) and three non-metastatic (MiaPaCa2, Panc-1 and ASPC-1). Firstly, microarray analyses showed that miR-100 is upregulated in metastatic cell lines compared to non-metastatic, and its upregulation corresponds to an increase in IGF-1R mRNA levels. Further Western blot analyses showed that IGF-1R is even seven-fold over-expressed in metastatic cells. Moreover, immunocytochemistry was used to examine a potential functional relationship between IGF-1R and miR-100. Cells transfected with a vector bearing an miR-100 inhibitor and GFP were compared to control cells without the inhibitor. The cells with the microRNA inhibitor expressed lower levels of IGF-1R, indicating that miR-100 upregulates the expression of IGF-1R, while the underlying mechanism remains unclear [56]. Recently, Lei et al. [57] identified that miR-7515 was downregulated in pancreatic cancer cells, with poor clinical outcomes, suggesting that miR-7515 might have a tumor-suppressive role in pancreatic cancer [57].

## 7. MicroRNA-Induced Regulation of IGF-1: Osteosarcoma

Osteosarcoma [58] and Ewing sarcoma [59] tissues have been investigated in the context of microRNA regulation of tumor progression and metastasis, as well as regarding the cells’ response to chemotherapy, associated with components of the IGF-1 pathway(s). Specifically, miR-16 was found to be downregulated in osteosarcoma cells compared to normal tissue, while over-expression of miR-16 in mice reduced cell proliferation and tumor progression. In addition, it was revealed that IGF-1R is a direct target of miR-16, and their connection was further confirmed in tissue samples, where higher levels of IGF-1R were associated with lower levels of miR-16. Hence, it has been speculated that miR-16 could be used for the suppression of tumorigenesis and uncontrolled cell proliferation in osteosarcoma [34]. A summary of the miRNAs, their targets and the associated conditions is shown in Table 1.

## 8. Future Perspectives and Clinical Applications

MiRNA-induced regulation of IGF-1 is a multifactorial, complicated system, importantly affecting cell physiology. Multiple roles of miRNAs in IGF-1’s effects on various types of cancer have been identified, and as miRNAs possess some unique properties, they could be used as therapeutic agents in cancer. Appropriately designed oligonucleotides can easily upregulate or downregulate specific miRNAs, while the latter provide easier delivery into target cells compared to large viral vectors [60]. Although miRNAs could be promising therapeutic agents, nevertheless, there are several challenges that should be solved, e.g., poor bioavailability and potential drug resistance [61]. Moreover, miRNAs could serve as possible diagnostic agents, as circulating miRNAs have been identified as potential biomarkers for early and easier detection of malignancies, e.g., for the detection of early-stage breast cancer by monitoring IGF-1 signaling [39].

Additional to the tumor-derived miRNAs that have significant clinical applications, circulating miRNAs, identified in blood, serum and plasma, are promising and non-invasive biomarkers for early cancer detection and monitoring [62,63,64,65]. These miRNAs are extracellularly viable by a wide range of mechanisms, such as encapsulation in exosomes, binding to RNA proteins or incorporation into lipoprotein complexes [63]. For example, circulating miRNA-143 has been found to be downregulated in colorectal cancer patients [64], while circulating miR-145 is widely accepted as a regulator across different types of cancers, and it has been detected in reduced levels in breast and liver cancer patients [65].

Future studies are needed to expand the current knowledge on the role of miRNAs in the regulation of other components of the IGF-1 bioregulation system and in various types of cancer, contributing further to the characterization of the role of miRNAs and their target genes as pathogenic, therapeutic and diagnostic molecules for cancer in clinical practice.

## 9. Conclusions

The control of the IGF-1/IGF-1R signaling pathway by microRNAs is essential for many physiological functions and is closely associated with the development, progression and response to treatment of several cancer types. In several tissues, including the breast, pancreas, liver, gastrointestinal tract, bone and muscle, it has been discovered that many microRNAs either directly or indirectly regulate the expression of IGF-1 and IGF-1R. In addition to affecting carcinogenesis, metastasis and cancer therapy resistance, these miRNAs may also function as biomarkers and therapeutic targets. Although most of the evidence comes from cell lines, several findings have been confirmed in patient tumor tissue or blood samples. For instance, in breast cancer tissue, reduced miR-122 and elevated IGF-1R were identified. Nevertheless, additional research, especially targeted clinical trials, on the relationships between miRNA and IGF-1 may lead to improved diagnostic methods and new targeted therapies for a range of cancer types.

## Figures and Tables

**Table 1:** A overview of the types of cancer, microRNAs (miRNAs), their sequences specified by strand (5p/3p), MIMAT accession numbers, targets and associated conditions.

Type of Cancer	miRNA	Strand/Sequence (5′–3′)	MIMAT ID	Target	Condition
Breast	MiR-126 [39]	3p/UCGUACCGUGAGUAAUAAUGCG	MIMAT0000445	IGFBP-2	Metastasis
	MiR-122 [38]	5p/UGGAGUGUGACAAUGGUGUUUG	MIMAT0000421	IGF-1R	Cancer biomarker
	miR-375 [40]	3p/UUUGUUCGUUCGGCUCGCGUGA	MIMAT0000728	IGF-1R	Resistance to therapy
	miR-145 [42]	5p/GUCCAGUUUUCCCAGGAAUCCCU	MIMAT0000437	IGF-1R	Cancer biomarker
Colorectal	miR-195 [41]	5p/UAGCAGCACAGAAAUAUUGGC	MIMAT0000461	IGF-1R	Cancer biomarker
	miR-497 [41]	5p/CAGCAGCACACUGUGGUUUGU	MIMAT0002874	IGF-1R	Sensitivity to apoptosis
	miR-143 [44]	3p/UGAGAUGAAGCACUGUAGCUC	MIMAT0004599	IGF-1R	Cancer staging
	miR-223 [35]	3p/UGUCAGUUUGUCAAAUACCCCA	MIMAT0000280	IGF-1R	Inflammatory bowel disease
	miR-139 [45]	5p/UCUACAGUGCACGUGUCUCCAGU	MIMAT0000250	IGF-1R	Metastasis
	miR-145 [46]	5p/GUCCAGUUUUCCCAGGAAUCCCU	MIMAT0000437	IGF-1R	Cancer biomarker
Gastric	miR-7 [43]	5p/UGGAAGACUAGUGAUUUUGUUGU	MIMAT0000252	IGF-1R	Cancer biomarker
Lung	miR-486 [48]	5p/UCCUGUACUGAGCUGCCCCGAG	MIMAT0002177	IGF-1R	Cancer biomarker
	miR-335 [49]	5p/UCAAGAGCAAUAACGAAAAAUGU	MIMAT0000765	IGF-1R	Metastasis
Hepatocellular	miR-145 [50]	5p/GUCCAGUUUUCCCAGGAAUCCCU	MIMAT0000437	IGF-1R	Cancer biomarker
	miR-190b [52]	5p/UGAUAUGUUUGAUAUUUGGGGU	MIMAT0004929	IGF-1	Insulin resistance
Pancreatic	miR-7 [54]	5p/UGGAAGACUAGUGAUUUUGUUGU	MIMAT0000252	IGF-1R	
	miR-139 [54]	5p/UCUACAGUGCACGUGUCUCCAGU	MIMAT0000250	IGF-1R	
	miR-145 [46]	5p/GUCCAGUUUUCCCAGGAAUCCCU	MIMAT0000437	IGF-1R	
	miR-1 [54]	3p/UGGAAUGUAAAGAAGUAUGUAU	MIMAT0000416	IGF-1R	
	miR-497 [55]	5p/CAGCAGCACACUGUGGUUUGU	MIMAT0002874	IGF-1R	Cancer biomarker
	miR-100 [56]	5p/AACCCGUAGAUCCGAACUUGUG	MIMAT0000098	IGF-1R	Unknown mechanism
Osteosarcoma	miR- 16 [34]	5p/UAGCAGCACGUAAAUAUUGGCG	MIMAT0000069	IGF-1R	Tumor progression

## Data Availability

No new data were created or analyzed in this study. Data sharing is not applicable to this article.
